# Effect of Rice Cultivation Systems on Indigenous Arbuscular Mycorrhizal Fungal Community Structure

**DOI:** 10.1264/jsme2.ME13011

**Published:** 2013-05-29

**Authors:** Nantida Watanarojanaporn, Nantakorn Boonkerd, Panlada Tittabutr, Aphakorn Longtonglang, J. Peter W. Young, Neung Teaumroong

**Affiliations:** 1 School of Biotechnology, Institute of Agricultural Technology, Suranaree University of Technology, Nakhon Ratchasima 30000, Thailand; 2 Department of Biology, University of York, PO Box 373, York YO10 5YW, UK

**Keywords:** arbuscular mycorrhizal fungi, compost application, cultivation systems, *Oryza sativa*, terminal restriction fragment length polymorphism (T-RFLP)

## Abstract

Arbuscular mycorrhizal fungi (AMF) in an agricultural ecosystem are necessary for proper management of beneficial symbiosis. Here we explored how the patterns of the AMF community in rice roots were affected by rice cultivation systems (the system of rice intensification [SRI] and the conventional rice cultivation system [CS]), and by compost application during growth stages. Rice plants harvested from SRI-managed plots exhibited considerably higher total biomass, root dry weight, and seed fill than those obtained from conventionally managed plots. Our findings revealed that all AMF sequences observed from CS plots belonged (only) to the genus *Glomus*, colonizing in rice roots grown under this type of cultivation, while rice roots sown in SRI showed sequences belonging to both *Glomus* and *Acaulospora*. The AMF community was compared between the different cultivation types (CS and SRI) and compost applications by principle component analysis. In all rice growth stages, AMF assemblages of CS management were not separated from those of SRI management. The distribution of AMF community composition based on T-RFLP data showed that the AMF community structure was different among four cultivation systems, and there was a gradual increase of Shannon-Weaver indices of diversity (*H*′) of the AMF community under SRI during growth stages. The results of this research indicated that rice grown in SRI-managed plots had more diverse AMF communities than those grown in CS plots.

Rice (*Oryza sativa* L.) is one of the important crops grown extensively in many countries. Generally, rice is grown under shallow flooded or wet paddy conditions, but it is also cultured where floodwater may be several meters deep; therefore, rice appears to have a high water requirement. Another system for rice cultivation, the system of rice intensification (SRI), originated in Madagascar in the early 1980s. SRI is composed of a set of practices that are used to cultivate rice, and these practices have now been adopted in over 20 countries. There are five main differences in paddy management when using SRI compared to conventional practices that are managed under wet paddy conditions or several meters deep floodwaters. These are: (i) transplanting younger seedlings, (ii) transplanting seedlings singly, (iii) using wider spacing, (iv) alternating soil flooding with draining during the vegetative growth phase, and (v) applying compost rather than mineral fertilizer. Increases in rice yields from 2 t ha^−1^ in conventionally managed paddies to 15–20 t ha^−1^ or higher for SRI-managed paddies have been reported (36). The mechanisms responsible for the marked yield increases obtained from these changes in management practices are still unclear. Previous studies have demonstrated that increased root growth (8), the addition of compost, and the presence of nitrogen-fixing bacteria and nitrifying bacteria in the rice rhizosphere (27, 31) are factors in the increased yields observed in the SRI system. Cycling of mineral nutrients, particularly nitrogen (N), is strongly affected by oxygen (O_2_) concentrations; hence, alternating wetting and drying of the soil are likely to strongly influence nutrient availability (16).

Rice plants readily form mycorrhizal associations under upland conditions, but under submerged conditions infection is rare due to the anoxic environment (12). Regardless, AMF are obligate aerobes in nature but can survive under waterlogged conditions, and this is supported by the fact that AMF as *Glomus etunicatum* showed fairly high colonization in rice roots and best survival under submerged conditions (3). Some reports have also revealed that inoculation of AMF in both high- and low-fertility soil could promote the nutrient acquisition of rice and increase rice yield under flooded conditions (8, 28). In the literature, there are insufficient studies providing an overview of the colonizing AMF in rice roots grown under SRI and there is still no clear picture of how the association may be exploited to benefit crop yield directly in fields. Nevertheless, this SRI system would create aerobic conditions in the soil that stimulate the colonization of rice roots by AMF and other fungi. Thus, the aims of this study were to explore how the patterns of AMF community structures and diversities in rice root were affected by rice cultivation systems (the SRI and the conventional rice cultivation system [CS]), by different growth stages, and by compost application.

## Materials and Methods

### Field sites and rice cultivation systems

The experimental field is located in the Northeastern region, Nakhon Ratchasima (lat. 102° 10′ E and long. 14° 97′ N), Suranaree University of Technology, Thailand. The study area is paddy soil, with mean annual rainfall of 906 mm (2010), and mean annual minimum and maximum temperatures of 27°C and 36°C, respectively. The soil is a sandy clay loam, pH 7.39, that contains (dry weight basis) 6.9 g kg^−1^ organic matter, 30.2 mg kg^−1^ P (BrayII; 9), 3 g kg^−1^ total N, 3.8 mg kg^−1^ NO_3_^−^, 1.9 mg kg^−1^ NH_4_^+^ and 89.5 mg kg^−1^ K (1 M NH_4_OAc, pH 7.0; 5). Field plots were arranged by a factorial approach in a completely randomized design (two factors; 2×2 factorial CRD) with three replications (4 treatments in 12 field plots). The experiment was designed with two factors that consisted of (i) a cultivation system (CS and SRI) and (ii) compost inoculation. Control treatments (no compost inoculation) were also tested to compare with treatments inoculated with compost. Compost was made from a mixture of agricultural waste, such as cassava peel, filter cake, chicken dung and cow dung. Compost (in dry matter, 8.8 g kg^−1^ N, 208 mg kg^−1^ NH_4_^+^, 1.3 g kg^−1^ NO_3_^−^, 39 g kg^−1^ P_2_O_5_, 9 g kg^−1^ K_2_O, pH 7.10 with moisture content 35%) was applied to both cultivation systems (except for control) at 12.5 t ha^−1^ (wet weight) seven days before rice seedlings were transplanted into the plots. Plots measured 8 m×8 m and were separated from each other by bunds. Rice was grown under two types of water management, the conventional flooded system (CS) and SRI. Water level was maintained every day 30 cm above the soil surface under CS conditions throughout rice growth stages, while the SRI had a 7–10 d interval with 3–5 cm water above the soil surface only during tillering to the flowering stage. The rice was planted in nursery beds (containing sterilized sand) at the beginning of February 2010 and seedlings were transplanted to conventional and SRI-managed plots at 30 and 15 days old, respectively. Plant spacing of 30 cm×30 cm with a single plant per hill was used in SRI, whereas three plants per hill with a plant spacing of 20 cm×20 cm were grown in CS. In SRI, 4–5 d of flooding were alternated with 4–5 d of draining during the vegetative growth period, while rice plots in the conventional system were kept flooded.

### Determination of AMF colonization and amount of P uptake

Rice plants were sampled four times at monthly intervals during the dry season (March to June 2010). The stages of rice development at each sampling time were: (i) vegetative growth phase after the first draining of SRI plots (March), (ii) vegetative growth phase one month after the previous sampling (April), (iii) rice panicle initiation (May), and (iv) rice harvest (June). Three rice plants were harvested from each plot in an area of 1 m^2^ (1 m×1 m). Roots of each individual were collected by carefully untangling them while briefly immersed in water. Half of the roots were stored in 95% ethanol for analysis of AMF colonization rates and half were dried at 50°C for 16 h and frozen at −80°C for DNA extraction. For colonization, roots were stained with trypan blue and all AMF structures were scored using the line intersect method at ×400 with 100 intersections (23). Shoots of each plant were oven dried at 70°C for 48 h, and ground. Ground plants were digested in acid mixture (HNO_3_+HClO_4_) for P analysis, and P concentration was analyzed by the vanadomolybdate blue method (39). Two-way measures ANOVA was used to analyze differences among treatment groups. The data were analyzed by factorial ANOVA using the Tukey-Kramer method (SPSS software ver. 17.0). Significant differences between means were established at *P*≤0.05.

### Components of yield

Rice plants were harvested from each plot in an area of 4 m^2^ (2 m×2 m between the bunds, with border rows on all sides). Grain, straw, and root were dried at 70°C for 48 h and weighed. Grain yield, root dry weight, and total above-ground biomass were calculated at 14% moisture content. All data were assessed by the factorial ANOVA approach in CRD using the Tukey–Kramer method (SPSS ver. 17.0). Significance of differences was established at *P*≤0.05.

### DNA extraction and T-RFLP reaction

Dried roots were ground in a microcentrifuge tube using a 4-mm glass bead in a Mixermill MM301 (Retsch, Haan, Germany) at 24 Hz, for 10 min. DNA of 20 mg ground roots was extracted using a DNeasy Plant Mini kit (Qiagen, Valencia, CA, USA) following the manufacturer’s instructions. DNA extracts were diluted 10× in PCR-grade water before PCR reactions (see below). A ~800-bp fragment of 18S rRNA gene was amplified by using the following primers: AMF-specific primer, AML1 and AML2 (19). In separate PCR reactions, untagged PCR products were created for cloning and sequencing, and tagged PCR products intended for T-RFLP. PCR reaction mixtures were performed using 0.2 mM of each dNTP, 10 pmol of each primer AML1 and AML2, 0.725 U *Taq* polymerase, and the supplied reaction buffer (Promega, Madison, WI, USA) in a final volume of 20 μL. PCR was run on a PTC100 (MJ Research, Waltham, MA, USA) under the following conditions: 95°C for 3 min, followed by 30 cycles of 94°C for 30 s, 63°C for 45 s, 72°C for 50 s, and extension step at 72°C for 5 min. PCR products were loaded onto 1% agarose gel and examined under UV light after staining with SYBR safe (Invitrogen, Carlsbad, CA, USA). Amplified products (~800 bp) were purified with a QIAquick PCR purification kit (Qiagen) for ligation and cloning.

For the T-RFLP reaction, AMF-specific primer pairs AML1-AML2 were labeled at both ends with different fluorescent labels (HEX-AML1 and FAM-AML2; Invitrogen). PCR mixtures and thermal cycling were performed as described above, except that the PCR-TRFLP was run for 20 cycles. The amplified PCR products were digested with the restriction enzyme having specific recognition sequences. The 3U of the restriction endonuclease *Hin*fI (Promega, Madison, WI, USA) were applied for digestion in a 20 μL reaction volume (three parallel reactions for each). Purified products were digested at 37°C for 3 h. Digestion reactions were further purified with QIAquick PCR purification kit (Qiagen) and eluted in 12 μL nuclease-free distilled water (Invitrogen). Samples were prepared with 6.5 μL deionized formamide, 0.5 μL of 600 LIZ internal size standard (Applied Biosystems, Foster City, CA, USA), and 3 μL *Hin*fI digested products. Prior to the T-RFLP run, the mixtures were denatured at 94°C for 5 min and immediately placed on ice for a few minutes. Fluorescently labeled terminal restriction fragments (T-RFs) were separated on an ABI PRISM 3730 DNA Analyzer Sequencer (Applied Biosystems), with three replicates for each digestion mixture to ensure reproducibility. GeneMapper version 3.7 software (Applied Biosystems) was used to analyze the labeled T-RF sizes and T-RF quantities (peak heights as rfu; relative fluorescent unit) by comparison with the internal size standard. Peak heights and peak areas were recorded with a minimum peak amplitude threshold of 50 rfu. For cases in which the strongest peaks on the electropherogram were not in the range 2,000–8,000 rfu, the sample concentration was adjusted accordingly and the sample was rerun.

### Cloning, sequencing, and phylogenetic analysis

A clone library was generated using pooled amplicons from three replicates of the same plot (3 plot replicates were carried out for a sample). Pooled PCR products were gel-purified to improve the efficiency of the ligation reaction (QIAquick gel purification kit; Qiagen). Purified products were ligated into the pGEM-T-Easy vector system cloning kit according to the manufacturer’s instructions (Promega) and transformed into *Escherichia coli* (JM109). Ninety-six positive transformants were selected randomly. Amplified inserts were then digested by the restriction enzyme *Hin*fI. Distinct RFLP profiles containing one or more clones were considered to be a distinct species. Each pattern of RFLP was screened and sequenced to ensure sequence identity with both directions of vector primers SP6 and T7 on ABI PRISM 3730 (Applied Biosystems) DNA Analyzer System at Macrogen (Seoul, South Korea). All sequences were compared in the public database by BLASTN searches and fifteen AMF sequences (phylotypes) were deposited in GenBank (accession numbers: JF906731–JF906744, JF906749). To separate AMF sequences from non-target sequences, the BLAST (2) was used. The resulting sequences (GenBank) were subjected to phylogenetic analysis using CLUSTALX version 1.83.1 (13), together with 46 reference sequences, and 3 sequences of other fungi were used as the outgroup. AMF sequences were automatically aligned, their closest BLAST matches, and additional AMF sequences from a phylogenetically representative set of *Glomeromycota* downloaded from GenBank. A neighbor-joining distances tree was constructed using MEGA version 4 (34) with 1,000 bootstrap replicates. All sequences were subjected to *in silico* restriction digestion with the program BioEdit 7.0 (9) to predict the fragment sizes of each double-labeled sequence fragment. Using this procedure, predicted fragment lengths were calculated, taking the differences in migration caused by dye properties (HEX vs FAM) into account. These *in silico* RFLP analyses were performed to putatively assign the most common T-RF pairs to specific sequences (AMF types). This procedure was only performed for T-RF pairs that were specific to a certain sequence. Related sequences having approx. 2 bp differences in T-RF sizes were considered to be the same, and this criterion was also used to match the observed T-RFs to sequences.

### T-RFLP analysis and diversity analysis

For T-RFLP runs, the threshold for peak recognition was set at 50 rfu. The resulting profiles were subjected to the following procedure: (i) exclusion of peaks <50 bp; (ii) size binning of peaks within a size range of 1.2 bp, and assignment to the maximal peak size to account for an incorporation; (iii) peak area calculation within the bins; peaks contributing <1% to the total area were excluded to achieve the same sensitivity across runs with different total fluorescence. The relative abundance was calculated by dividing the individual T-RF peak area attributable to a given species by the total fluorescence in the profile.

To evaluate richness and evenness, diversity statistics were calculated using the number and area of peaks in each average profile as representations of the number and relative abundance of different phylotypes in a sample. AMF community diversity was calculated by three replicate T-RFLP profiles generated from each replicate of root sample (3 replicates of each sample). The TRF signal in the simulated T-RFLP profile (representing peak area) was assumed to be proportional to the relative abundance of the species. TRF signals were pooled when two species had the same TRF size in base pairs. TRFs <50 bp and >600 bp were deleted, and TRFs were expressed as the relative abundance of the total signal detected in the T-RFLP profile. TRFs below a threshold (<1% of the total profile signal) were also deleted, and the relative abundance of the remaining TRFs was recalculated. Phylotype richness (*S*) was calculated as the total number of distinct TRF sizes (between 50 and 600 bp) in a profile. The Shannon-Weiner diversity index (*H*′) was calculated as follows: *H*′=−∑(*p_i_*) (log_2_ *p_i_*), where *p_i_* is the proportion of an individual peak area relative to the sum of all peak areas. Evenness was calculated from the Shannon-Weiner diversity function: *E*=*H/H*_max_, where *H*_max_=log_2_(S) (21).

### Data and statistical analysis

Principle component analysis (PCA) was performed to compare the AMF communities in rice roots under different cultivation systems and compost inoculation during four growth stages, using the binary data of the AMF phylotype (based on clone library; presence/absence of AMF sequence) of each field plot as input data. PCA was executed for all samples together and carried out using the SPSS version 17.0 software package. For the purpose of the analysis, each distinct RFLP pattern profile was considered to be a distinct operational taxonomic unit (OTU). Since the sample size was required to adequately represent the real population, rarefaction curves of observed OUTs were generated using the data set from 18S rRNA gene clone libraries. Rarefaction curves were calculated by EcoSim ver. 7.71 (Acquired Intelligence, Kesey-Bear), for pooled data (the 1^st^–4^th^ sampling times) and for each sampling time, and the confidence interval for curves was set at 95%. To visualize the differences among AMF communities determined by T-RFLP analysis, the TRF peak area values were used as input data for multidimensional scaling in two dimensions using the ALSCAL in the SPSS version 17.0 software package.

## Results

### AMF colonization and P uptake

The results revealed that variations in AMF colonization and P uptake among different growth stages and compost inoculation. Statistical analysis demonstrated interactions in AMF colonization and P uptake between growth stages (sampling times) and compost inoculation (treatments) (*P*≤0.01). The growth stages, 90 and 120 d, provided the greatest AMF colonization rates of all treatments. For compost inoculation (treatments), it was found that rice plants grown under SRI cultivation and inoculated with compost had a higher colonization rate than other treatments in all growth stages. At 90 and 120 d of growth stage, rice plants grown in SRI and amended with compost significantly showed the highest percentage of colonization, followed by control plants grown in SRI, while the lowest colonization rates were detected in the first stage of control treatment and treatment amended with compost alone under CS management (Table 1). In the case of P uptake, the results indicated that there were interactions between sampling times and compost applications (treatments) (*P*≤0.01) (Table 2). At 90 d of growth stage, the best P uptake was produced in all treatments. Considering the treatments, rice plants sown in SRI and amended with compost exhibited the highest P uptake in all stages of rice growth.

### Rice yield

The rice plants harvested from the SRI-managed plots showed significantly higher total biomass, root dry weight, and seed fill than those obtained from CS plots (Table 3). There were no interactions of grain yield (*P*=0.420) and rice yield components (total biomass; *P*=0.488, root dry weight; *P*=0.912, seed fill; *P*=0.368, plant height main stem; *P*=0.696) between the plot management and compost application. Neither significant differences between the two water management systems nor between the compost-amended and unamended treatments were observed in grain yield and plant height main stem. Total biomass was significantly affected by plot management (*P*<0.01) but was not significantly affected by the compost applied (*P*=0.075). We found that rice plants grown in SRI plots provided higher total biomass than those sown in CS plots. The root dry weight was significantly affected by plot management (*P*=0.02) but was not significantly affected by compost application (*P*=0.835). Once again, seed fill appeared to be significantly influenced by plot management (*P*<0.01). Rice plants managed by SRI plots had significantly higher percentages of seed fill than those managed by CS plots in both compost-applied and unapplied treatments.

### Phylogenetic analyses of AMF groups

**Fig. 1:**
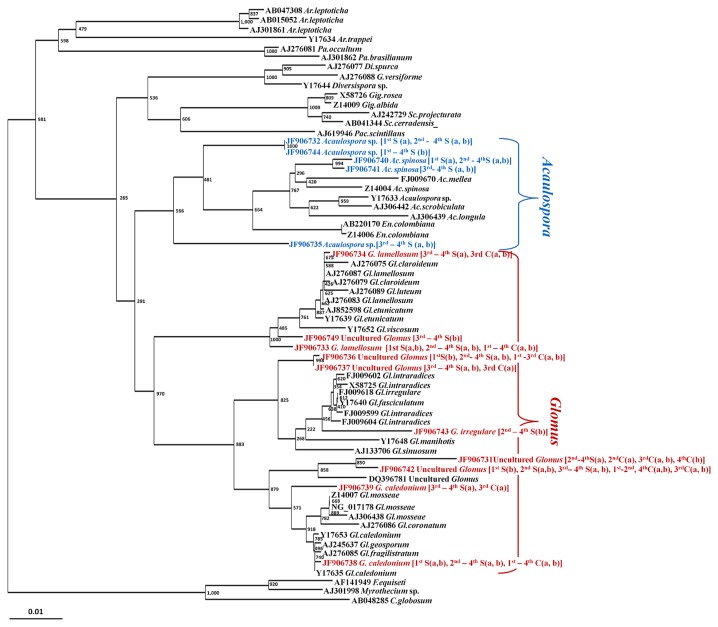
Phylogenetic relationships derived from partial 18S rRNA gene sequences (1,000 bootstrap) of AMF colonizing rice roots grown under control and compost treatments in two different rice cultivation systems (conventional cultivation system, CS and system of rice intensification, SRI). GenBank accessions with letters in parentheses indicated that AMF sequences obtained from rice roots, which were grown under conventional (C) and system of rice intensification (S) conditions and inoculated without (a) or with (b) compost treatments during the 4 growth stages (vegetative, 1^st^; tillering, 2^nd^; flowering, 3^rd^; and harvest, 4^th^), (*i.e.*, [1^st^ S(b)] indicates AMF colonizing the roots of rice collected at 1^st^ stage of growth, under SRI condition, and inoculated with compost). Letters in blue and red represent AMF sequences, which were derived from rice roots grown in the field plot experiment, belonging to the genera *Acaulospora* and *Glomus*, respectively.

All sequences had high levels of similarity (98% to 100% identity) to AMF sequences, belonging to members of the phylum *Glomeromycota*, the genera *Glomus*, and *Acaulospora*. No other AMF genera appeared in any samples, although the AMF-specific primers (AML1-AML2) used in this study could also amplify 18S rRNA gene of other AMF (the phylum *Glomeromycota*). The constructed alignment included 46 different glomalean sequences and 3 other fungal sequences defined as the outgroup. Some clones producing the same sequence were represented just once in the alignment. The sequences obtained in this study were clustered into fifteen discrete groupings or fungal sequence types. Ten of these sequences belonged to the genus *Glomus*, which was the most frequently obtained from rice root DNA. Another group of the 5 AMF sequences belonged to the genus *Acaulospora*. The phylogenetic tree demonstrated that all *Glomus* and *Acaulospora* sequences from the present study, as expected, were grouped into their own genera (Fig. 1). Our results revealed that all AMF sequences obtained from the rice roots in CS plots belonged only to the genus *Glomus*, and no *Acaulospora* sequences appeared in rice grown with this cultivation method. In other words, the rice roots sown under SRI showed AMF sequences that belonged to both AMF genera, *Glomus* and *Acaulospora*.

### Comparisons of AMF communities and AMF distribution

**Fig. 2:**
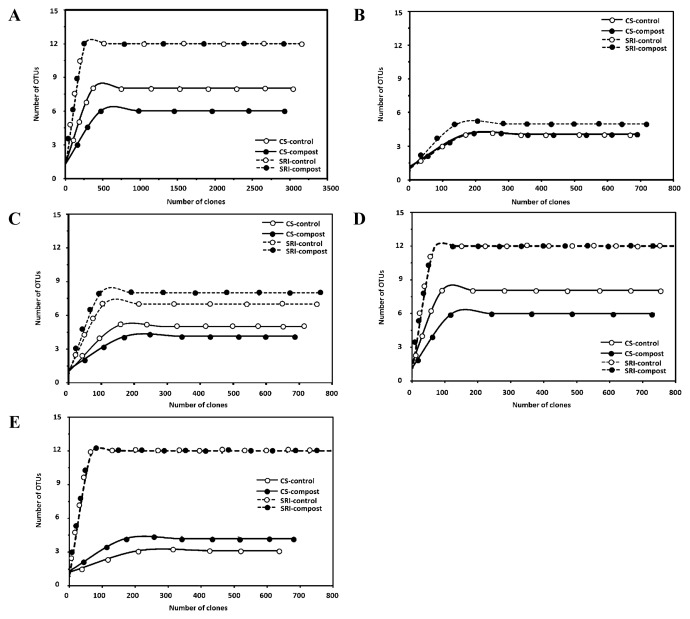
Rarefaction curves of observed OUTs in the AM rice root 18S rRNA gene library, constructed from CS-control, CS-compost, SRI-control and SRI-compost plots, for the pooled sampling times (A), 1^st^ sampling time (B), 2^nd^ sampling time (C), 3^rd^ sampling time (D), and 4^th^ sampling time (E). Rarefaction curves were calculated with EcoSim at 95% confidence intervals (95% Cl).

Rarefaction curves were constructed for the pooled sampling times (Fig. 2A) and the 1^st^, 2^nd^, 3^rd^, and 4^th^ sampling times (Fig. 2B–E), in order to assess whether true species diversity was captured though RFLP analysis. Rarefaction curves demonstrated that true species diversity was likely captured for all clone libraries, since the curves reached a plateau at the maximum number of RFLP patterns, suggesting that the number of clones was adequate to estimate the diversity of AMF colonizing the rice root samples.

**Fig. 3:**
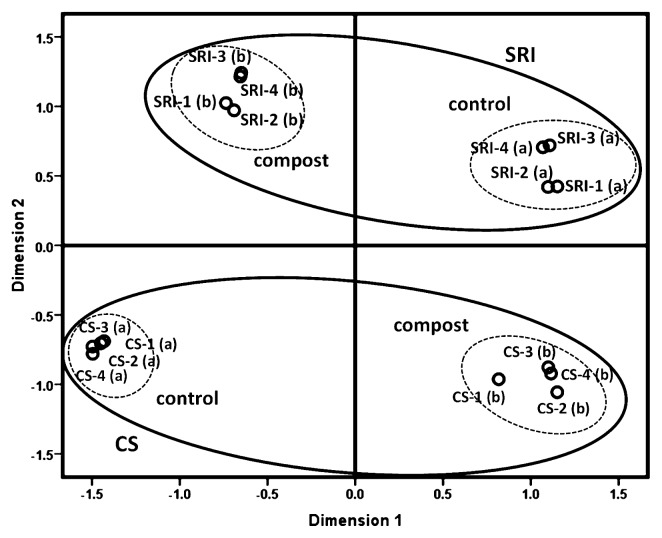
Multidimensional scaling (distances as interval; SPSS 17.0) plot of the AM fungal community composition found in the roots of rice plants grown under the two different cultivations, and the treatments of control and compost inoculations. The eigenvalues of the first and second axes in the two-dimensional ordination diagrams are as follows: dimension 1, 0.5; dimension 2, 0.4. Full oval and dashed oval represent the distributions of AM community in rice roots grown under SRI and CS plots, and applied with compost, respectively. The letters (a and b) indicate AMF community composition of rice roots under control and compost treatments, respectively, and the numbers (1, 2, 3 and 4) represent the first, second, third and last sampling times, respectively.

The distribution of AMF community composition (based on T-RFLP data) for all sampling times was given in the multidimensional scaling ordination, and a plot diagram (Fig. 3) displayed the AMF communities grouped clearly by the two water management systems and compost applications. Among different sampling times (different plant growth stage), AMF communities (derived from TRF-peak area data input) in the rice field plots revealed a shift in community composition. The AMF community structure associated with different cultivation plots (CS and SRI) and amended with compost was separated into four groups (*i.e.*, SRI-control, SRI-compost, CS-control and CS-compost).

### AMF community diversity

**Fig. 4:**
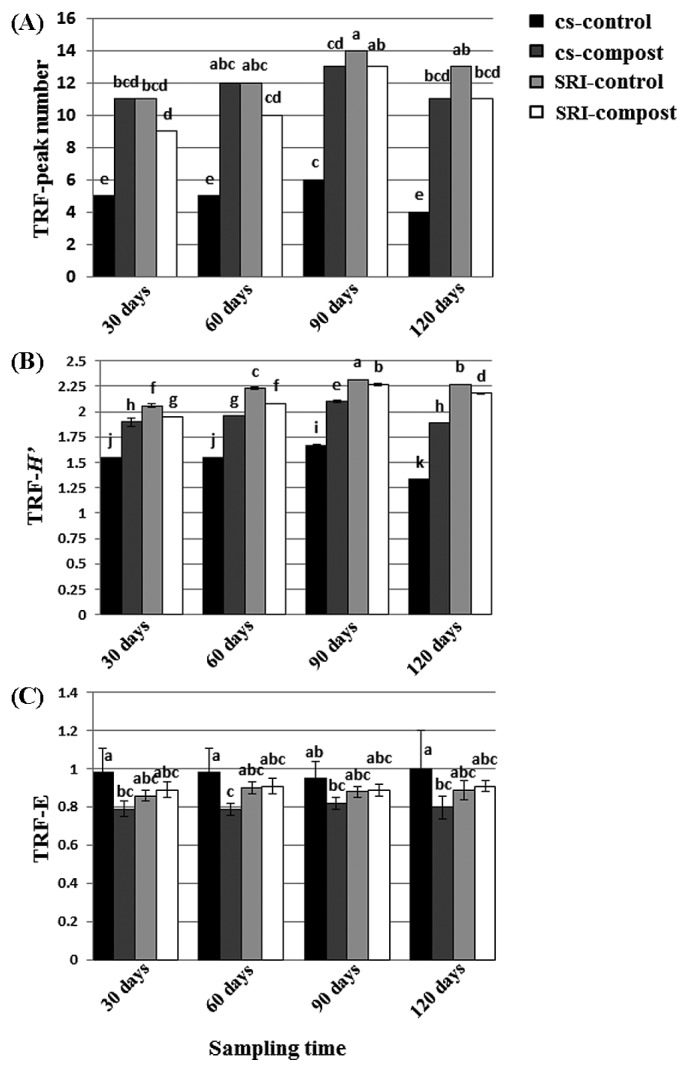
Diversity statistics calculated from T-RFLPs of AMF 18S rRNA gene amplified from rice root DNA indicate TRF peak number (A), TRF-*H*′ (B), TRF-E (C) at 4 stages of growth.

Diversity statistics were calculated using the T-RFLP profile (TRF indices) (Fig. 4). The results demonstrated interactions in TRF peak numbers and the *H*′ between growth stages and between treatments (*P*≤0.01), while species evenness (E) showed no interactions between the two factors (growth stages and treatments). There was no significant difference in evenness (E) among cultivation systems (CS and SRI) and among compost inoculations (control and compost treatments). The numbers of TRF peaks detected significantly differed between growth stages and between treatments. Ninety days of sampling provided the greatest number of TRFs peak in all treatments. There were variations in TRF peak numbers among different cultivations and compost applications. Rice plants control grown under SRI produced the highest peak numbers (14 peak numbers), followed by compost treatment grown in SRI and CS (13 peak numbers), and control treatment sown in an SRI plot (13 peak numbers), while control treatment grown in a CS plot provided the lowest peak numbers (4–6 peak numbers) at all sampling times. The *H*′ had a significant difference between growth stages and treatments, and varied between 1.33–2.32. The highest *H*′ was detected in a rice plant sampled at 90 d among all growth stages. Control rice plants (no compost inoculation) grown under SRI showed the highest value (*H*′=2.32) at 90 d, while the lowest value (*H*′=1.33) was detected in control plants grown in a CS plot at 120 d. Considering the first (30 d), second (60 d), third (90 d) and last (120 d) stages of growth, control plants (no compost inoculation) sown in SRI gave the most *H*′ (*H*′=2.06, 2.22, 2.32, and 2.27, respectively), while the lowest AMF diversity (*H*′=1.54, 1.55, 1.67, and 1.33, respectively) was found in control plants grown under CS management. Additionally, our study pointed out that rice grown in SRI showed higher *H*′ than rice plants grown under CS management. The results also revealed that there was an increase of *H*′ in rice plants sown under SRI during growth stages. The output indicated that rice plants grown under SRI had a more diverse AMF community than those grown under CS conditions.

## Discussion

Although wetland rice has previously been considered non-mycorrhizal, a positive response to AMF inoculation has been observed (29). Our results presented here (Table 1) showed that AMF associated in rice roots could colonize under waterlogged conditions (anaerobic conditions), and there was an increase of the AMF colonization rate over the sampling times. Solaiman and Hirata (30) also reported that 28% colonization by *Glomus* spp. at six weeks of growth (after growing in a dry nursery) persisted in a wetland. Although AMF is an obligate aerobic in nature, it can probably survive in association with rice roots under anaerobic conditions because it obtains O_2_ from the atmosphere through rice aerenchymatous tissue. It was found in this study that AMF diversity differed among cultivation treatments. The changes in AMF diversity in response to the different cultivation systems and the application of compost might be due to several factors, such as pH, nutrient content, total soil C and N, and temperature, which are known to influence AMF distribution (11). In addition, organic matter addition was able to increase AMF hyphae growth (17, 14). Sooksa-nguan and colleagues (32) also revealed changes in microbial communities in which differences in rice cultivation systems affected the structures of *Bacteria* and *Archaea* communities. This suggest that the SRI practice, where flooding was alternated with periods of draining, allowed for higher O_2_ availability in the soil with higher AMF establishment than conventionally managed practice, while the addition of compost might favor nutrients as well as being a growth condition that could induce diversity of AMF.

The high level of AMF diversity in SRI practice was correlated with a clear increase of the yield components, such as total biomass, root dry weight, and seed fill of rice grown under SRI management (Table 3). Similar observations of improved performance of rice yield with wider spacing under SRI have also been reported previously (18, 24). A previous report indicated that maintenance of a high CO_2_ assimilation rate via a delay in leaf senescence is an important factor that can increase the crop yield of SRI (6). Younger seedlings used with SRI perform better in terms of various root characteristics (root length density and root weight density) than older seedlings (25). SRI water management practices also help in improving root systems (4), while continuous flooding can cause degeneration of as much as three-fourths of roots by the flowering stage (15). In addition, the high level of AMF colonization (Table 1) was most likely correlated with high P uptake in rice plants grown under SRI management (Table 2); however, some treatments did not show a correlation between AMF colonization and high P uptake. For example, at 30 d, AMF colonization under CS control treatment was lower than that in SRI-control, but P uptake in CS-control was higher than in SRI-control. It is possible that rather than AMF colonization, other factors were more effective in causing differences in P uptake among the treatments, such as the rice growth stage and compost treatment. Thus, diverse AMF colonization under SRI management most likely played a role in enhancing the P concentration and uptake, and thereby improving rice growth, showing that the enhancement of P uptake in response to AMF infection may have been the result of the increased absorption of nutrients due to the greater surface area of AMF hyphae extending from the roots. The outcome observed here, that the highest P uptake was detected at 90 d (Table 2), suggested that rice plants at the end of the panicle initiation stage may be undergoing full growth and demand large amounts of nutrients. Although the colonization of AMF was effective on P uptake and promoted rice yield components, the grain yields were not higher in SRI-managed rice than in CS plots. It is possible that the AMF species in these communities and their effectiveness may significantly affect nutrient availability and plant growth. Moreover, AMF may be more effective in the vegetative stage, rather than for reproductive growth. Our finding was similar to some upland rice-growing regions where rice yield was reduced in SRI-managed plots (26). Thus, AMF in SRI management may have a role in nutrient uptake into rice before plants enter the reproductive stage.

The majority of sequences detected in rice roots belonged to the genus *Glomus*. Other reports on the molecular diversity of AMF also showed that *Glomus* was the dominant genus in AMF communities in different ecosystems (7, 10, 40). The sequences clustering in the genera *Acaulospora* were found only in the plots managed under SRI, and were not detected under CS conditions. The occurrence of *Glomus* groups in rice roots grown in CS plots suggests that these fungi might be more tolerant of anaerobic conditions. As in previously reported studies carried out for other eco-systems such as tropical forests (11), agricultural sites (10), wetland soils (40), gypsum soils (1), or polluted soils (37), they also found an AMF community dominated by *Glomus* species. Nonetheless, it remains unknown why *Glomus* species are better adapted to disturbed environments. Further analyses are needed to confirm whether differences in water management may favor particular genera or species of AMF over others. It is also necessary to investigate whether any changes in species composition may be significantly affected by nutrient availability and plant growth since the AMF community differed not only by cultivation system, but also by the application of compost. Although two major AMF in the genera *Glomus* and *Acaulospora* were detected from rice roots with or without compost application (Fig. 1), the species diversity of these two genera may be different as PCA, which was derived from different RFLP patterns of AMF sequences obtained from the clone library exhibited a difference in AMF community composition between control and compost applications (Fig. 3). It was also found that *Glomus* (JF906743) and *Glomus* (JF906749) were only detected in S(b) treatments (Fig. 1). Moreover, the diversity of the AMF community was also the highest in SRI with compost treatment (Fig. 4). This result supported the hypothesis that the nutrient content or total soil C and N may influence AMF distribution, especially when rice was grown under SRI management that had more O_2_ available than in the conventional system. In addition, *Glomus* sp. tends to be easily detected more than other AMF genera, even in other environments. Thus, it is possible to detect many species of the genus *Glomus* compared with other genera.

Here, we questioned whether differences in diversity between communities can be meaningfully portrayed by common diversity indices applied to T-RFLP profiles derived from AMF communities. Our results demonstrated diversity estimates presented by TRF richness (number of TRFs in profile), *H*′ and evenness (frequency distribution of TRFs) of AMF communities derived from T-RFLP data. Interestingly, a high percentage of AMF colonization was observed for the high diversity of the AMF community in roots growing in SRI plots. SRI plots sampled at 90 and 120 d revealed the highest level of colonization and showed higher AMF diversity (based on *H*′ derived from SSU) than the other plots at 90 d (Table 1, Fig. 4 and also see Table S1). High AMF diversity was reported in the dry Afromontane forest (*H*′=2.58 based on ITS) and in a tropical forest (*H*′=2.33 based on SSU) (11). There was higher AMF diversity in the SRI plot, potentially because higher AMF diversity may reflect the high colonization of AMF in aerobic rice field plots.

T-RFLP can be applied as a quantitative method to estimate the relative abundance based on peak heights or peak areas (20). Analyses by multidimensional scaling ordination (Fig. 3) showed that PCA exhibited a difference in AMF community composition among cultivation managements and compost applications, and marked shifts in composition were detected by multidimensional scaling ordination based on abundance data. Moreover, in the results of the rarefaction curve, the expected number of AMF species in rice roots was higher in the plots of CS-control than in CS-compost (Fig. 2A), which showed the same trend as described in the phylogenic tree (Fig. 1). However, as the analysis described the T-RFLP peak number, it was implied that the diversity and species number of AMF was higher in CS-compost than in CS-control (Fig. 4A). Disagreement of the results suggests that the marked shifts in community compositions might have been biased by the PCR approach. The limitations of T-RFLP for quantitative characterization of communities have been well argued. These problems become more evident when working with complex ecosystems in which diversity of the microorganisms is high, with differences in the ability to extract DNA from different organisms, genome size, and G+C content (33, 22). Almost every step (PCR amplification or digestion or TRF run) of the T-RFLP technique can introduce biases or errors to the analysis (38). PCR amplification of a species is known to be directly influenced by the presence of other species in the PCR mixture, affecting the apparent abundance of a species in the PCR reaction. As a result of these biases, representation of the real microbial community and diversity in ecosystems will probably not be possible; however, the T-RFLP facilitates the separation of mixtures of PCR-amplified gene fragments based on terminal restriction fragments (T-RFs) and allows large numbers of samples to be analyzed simultaneously (35). Thus, this technique is still suitable for monitoring the changes in microbial communities in response to changes in environmental factors.

## Conclusions

Different cropping systems, alternating flooding and draining cycles of water management, affected AMF community structure in paddy fields. The results revealed that rice plants grown under SRI had a more diverse AMF community than those grown under CS conditions.

## Supplementary Material



## Figures and Tables

**Table 1:** Effect of compost inoculation under conventional cultivation systems (CS) and system of rice intensification (SRI) on mycorrhizal root colonization of a rice field plot at 30, 60, 90, and 120 d of growth stages

Sampling times	Treatments	AMF colonization (%)
30 days^c^	CS-control^d^	11.1±0.2 l†
	CS-compost^c^	10.2±0.2 m
	SRI-control^b^	20.7±0.2 gh
	**SRI-compost**^a^	**29.2±0.3 e**

60 days^b^	CS-control^d^	17.2±0.5 k
	CS-compost^c^	18.3±0.8 j
	SRI-control^b^	27.8±0.2 f
	**SRI-compost**^a^	**34.1±0.1 d**

**90 days**^a^	CS-control^d^	19.2±0.3 i
	CS-compost^c^	20.3±0.4 gh
	SRI-control^b^	35.5±0.3 c
	**SRI-compost**^a^	**40.0±0.2 a**

**120 days**^a^	CS-control^d^	20.1±0.4 h
	CS-compost^c^	20.8±0.7 g
	SRI-control^b^	37.0±0.5 b
	**SRI-compost**^a^	**39.8±0.3 a**

**	**	**

† Means under each parameter followed by the same letter are not significantly different (*P*≤0.05) according to the Tukey–Kramer method. Values are the means±standard errors calculated from three replicates.

‡ Bold indicates the highest value in AM colonization.

** Statistical level indicates significant differences among sampling times and treatments at *P*≤0.01.

**Table 2:** P uptake in rice plants grown under conventional cultivation systems (CS) and system of rice intensification (SRI) in rice field plot at 30, 60, 90, and 120 d of growth stages

Sampling times	Treatments	P uptake (g/plant)
30 days^b^	CS-control^b^	2.4±1.0 cd†
	CS-compost^b^	3.0±0.9 cd
	SRI-control^b^	1.0±0.5 e
	**SRI-compost**^a^	**3.3±2.5 bcd**

60 days^b^	CS-control^b^	3.7±1.0 bc
	CS-compost^b^	4.3±0.4 bc
	SRI-control^b^	2.3±1.5 cd
	**SRI-compost**^a^	**3.4±1.6 bcd**

**90 days**^a^	CS-control^b^	4.0±0.6 bc
	CS-compost^b^	4.3±1.5 bc
	SRI-control^b^	3.7±1.4 bc
	**SRI-compost**^a^	**6.9±1.9 a**

120 days^b^	CS-control^b^	2.0±0.7 cd
	CS-compost^b^	2.1±0.1 cd
	SRI-control^b^	4.3±0.4 bc
	**SRI-compost**^a^	**5.6±1.7 ab**

**	**	**

† Means under each parameter followed by the same letter are not significantly different (*P*≤0.05) according to the Tukey–Kramer method. Values are the means ± standard errors calculated from three replicates.

‡ Bold indicates the highest value in AM colonization.

** Statistical level indicates significant differences among sampling times and treatments at *P*≤0.01.

**Table 3:** Grain yield and yield components of rice from conventionally (CS)- and SRI-managed plots with and without compost application

Treatments	Grain yield (t ha^−1^)	Total biomass (t ha^−1^)*	Root dry weight (t ha^−1^)	Seed fill (%)**	Plant height main stem (cm)
Conventional (CS)					
Control	1.4±0.5 ns‡	2.7±0.9 b†	0.02±0.0 b	70.2±6.6 b	92.6±5.0 ns
Compost	1.6±0.1 ns	3.2±0.2 b	0.02±0.0 b	75.5±7.5 b	95.3±3.9 ns
SRI					
Control	1.7±0.0 ns	5.3±0.1 a	0.11±0.1 a	96.7±0.2 a	93.5±3.3 ns
Compost	1.7±0.2 ns	6.3±0.1 a	0.13±0.0 a	96.5±0.4 a	98.0±2.7 ns

† Means under each parameter followed by the same letter are not significantly different (*P*≤0.05) according to the Tukey–Kramer method. Values are the means ± standard errors calculated from three replicates.

‡ NS indicates no significant differences among treatments at *P*≤0.05.

* Total biomass=sum of total above ground plant dry weight and root dry weight.

** Seed fill=the ratio of good grain number to total grain number (good grain+loss grain)×100
