# Supplementary material for: Effect of Rice Cultivation Systems on Indigenous Arbuscular Mycorrhizal Fungal Community Structure
Source: Microbes Environ. 2013 May 29;28(3):316–24. doi: 10.1264/jsme2.ME13011 (PMC4070969; doi:10.1264/jsme2.ME13011)
Supplement: Supplementary file 1 [file 28_316_s1.pdf]

Table S1. Diversity statistics calculated from T-RFLPs of AMF 18S rDNA amplified from rice root DNA

**Sampling times**	**Treatments**	**Diversity indices**		
		**TRF-S***	**TRF-*H*′**	**E**
30 days	CS-control	5.0 ± 0.0e†	1.55 ± 0.00j	0.98 ± 0.13a
	CS-compost	11.0 ± 0.0bcd	1.90 ± 0.04h	0.79 ± 0.04bc
	SRI-control	11.0 ± 0.0bcd	2.06 ± 0.02f	0.86 ± 0.03abc
	SRI-compost	9.0 ± 0.0d	1.95 ± 0.00g	0.89 ± 0.04abc
60 days	CS-control	5.0 ± 0.0e	1.55 ± 0.00j	0.98 ± 0.13a
	CS-compost	12.0 ± 0.0abc	1.96 ± 0.00g	0.79 ± 0.03c
	SRI-control	12.0 ± 0.0abc	2.23 ± 0.01c	0.90 ± 0.03abc
	SRI-compost	10.0 ± 0.0cd	2.08 ± 0.00f	0.91 ± 0.04abc
**90 days‡**	CS-control	6.0 ± 0.0e	1.67 ± 0.01i	0.95 ± 0.09ab
	CS-compost	13.0 ± 0.0ab	2.10 ± 0.01e	0.82 ± 0.03bc
	SRI-control	**14.0 ± 0.0a**	**2.32 ± 0.00a**	0.88 ± 0.03abc
	SRI-compost	13.0 ± 0.0ab	2.27 ± 0.01b	0.89 ± 0.03abc
120 days	CS-control	4.0 ± 0.0e	1.33 ± 0.00k	1.00 ± 0.20a
	CS-compost	11.0 ± 0.0bcd	1.89 ± 0.00h	0.80 ± 0.06bc
	SRI-control	13.0 ± 0.0ab	2.27 ± 0.00b	0.89 ± 0.05abc
	SRI-compost	11.0 ± 0.0bcd	2.18 ± 0.01d	0.91 ± 0.03abc

† Means under each parameter followed by the same letter are not significantly different ($P \leq 0.05$) according to the Tukey–Kramer method. Values are means± standard errors calculated by three replicates.

‡ Bold face type indicates the highest diversity indices among all sampling times.

* Means are calculated by each column.
